# Effects of Organic Phosphorus on Methylotrophic Methanogenesis in Coastal Lagoon Sediments With Seagrass (*Zostera marina*) Colonization

**DOI:** 10.3389/fmicb.2020.01770

**Published:** 2020-07-31

**Authors:** Shiling Zheng, Bingchen Wang, Gang Xu, Fanghua Liu

**Affiliations:** ^1^Key Laboratory of Coastal Biology and Biological Resources Utilization, Yantai Institute of Coastal Zone Research, Chinese Academy of Sciences, Yantai, China; ^2^Laboratory for Marine Biology and Biotechnology, Pilot National Laboratory for Marine Science and Technology, Qingdao, China; ^3^Key Laboratory of Coastal Environmental Processes and Ecological Remediation, Yantai Institute of Coastal Zone Research, Chinese Academy of Sciences, Yantai, China; ^4^College of Environment and Safety Engineering, Qingdao University of Science and Technology, Qingdao, China; ^5^Guangdong Key Laboratory of Integrated Agro-environmental Pollution Control and Management, National-Regional Joint Engineering Research Center for Soil Pollution Control and Remediation in South China, Guangdong Institute of Eco-environmental Science and Technology, Guangdong Academy of Sciences, Guangzhou, China

**Keywords:** organic phosphorus, methylotrophic methanogens, seagrass vegetated regions, *Methanolobus*, *Methanosarcina*

## Abstract

Methanogens are the major contributors of greenhouse gas methane and play significant roles in the degradation and transformation of organic matter. These organisms are particularly abundant in Swan Lake, which is a shallow lagoon located in Rongcheng Bay, Yellow Sea, northern China, where eutrophication from overfertilization commonly results in anoxic environments. High organic phosphorus content is a key component of the total phosphorus in Swan Lake and is possibly a key factor affecting the eutrophication and carbon and nitrogen cycling in Swan Lake. The effects of organic phosphorus on eutrophication have been well-studied with respect to bacteria, such as cyanobacteria, unlike the effects of organic phosphorus on methanogenesis. In this study, different sediment layer samples of seagrass-vegetated and unvegetated areas in Swan Lake were investigated to understand the effects of organic phosphorus on methylotrophic methanogenesis. The results showed that phytate phosphorus significantly promoted methane production in the deepest sediment layer of vegetated regions but suppressed it in unvegetated regions. Amplicon sequencing revealed that methylotrophic *Methanococcoides* actively dominated in all enrichment samples from both regions with additions of trimethylamine or phytate phosphorus, whereas methylotrophic *Methanolobus* and *Methanosarcina* predominated in the enrichments obtained from vegetated and unvegetated sediments, respectively. These results prompted further study of the effects of phytate phosphorus on two methanogen isolates, *Methanolobus psychrophilus*, a type strain, *Methanosarcina mazei*, an isolate from Swan Lake sediments. Cultivation experiments showed that phytate phosphorus could inhibit methane production by *M. psychrophilus* but promote methane production by *M. mazei*. These culture-based studies revealed the effects of organic phosphorus on methylotrophic methanogenesis in coastal lagoon sediments and improves our understanding of the mechanisms of organic carbon cycling leading to methanogenesis mediated by organic phosphorus dynamics in coastal wetlands.

## Introduction

Coastal wetlands are considered “sinks,” “sources,” and “transformers” of phosphorus, with important regulatory effects on offshore marine environments (Nixon and Lee, 1986; Nixon et al., 1996; Istvánovics, 2001). As an important phosphorus source, organic phosphorus (OP) is a product of the combination of P and organic groups, and its common source is organic matter, for example, monoester phosphorus, diester phosphorus, and phytate phosphorus. OP mineralization significantly supplements dissolved inorganic phosphorus (DIP), a key limiting factor for biological growth. This phosphorus transformation in sediments is a vital endogenous cause of inshore eutrophication (Schindler et al., 2008). However, there is limited knowledge about the effects of OP transformation on biogenic methane formed by methanogenic microorganisms in coastal sediments.

Methanogenic microorganisms are important carbon cycle participants in coastal areas and are an important part of the biogeochemical cycle and as contributors to environmental health (Christensen et al., 2018). In general, in sulfate-reducing zones of marine sediments, hydrogenotrophic and acetoclastic methanogenesis are inhibited by competition for available electron donors such as the hydrogen and acetate consumed by sulfate-reducing bacteria (SRB) (Broman et al., 2017). Among different substrates utilized by methanogens, marine methylated compounds, such as methanol and mono-, di-, or trimethylamines, are major substrates for methane production by methylotrophic methanogens. The primary sources of methylated compound substrates are the microbial decomposition of marine organisms or the root exudates of plants such as mangroves, *Spartina alterniflora* Loisel, and seagrasses (Mohanraju and Natarajan, 1992; Lyimo et al., 2009; Sun et al., 2015; Yang et al., 2019; Zheng et al., 2019). These substrates are not routinely used by SRB in the presence of methanogens (Lyimo et al., 2009), but promote the rapid growth of methylotrophic methanogens in sediments with high densities of seaweed. As a result, methylotrophic methanogenesis may be a significant source of methane in coastal ecosystems covered by vegetation.

As a crucial transitional site between sea, river, and land, Swan Lake (SL) is subjected to anthropogenic influences and biological precipitation, resulting in high concentrations of OP in sediments, accounting for 25–40% of total phosphorus (Zhang et al., 2017). SL, a naturally occurring lagoon, directly connects to the Yellow Sea (YS) via a narrow inlet and contains a large area of *Zostera marina* seagrass meadow. With increasing aquaculture, agriculture, and domestic pollution, the inlet was overwhelmed with frequent algal blooms, leading to a functional deterioration of SL wetlands (Shao et al., 2015). As a result, the top layer, sulfate-reducing zone absorbed substantial organic matter and the deeper transition layer and deepest methanogenic layer were also influenced. In recent years, many studies have been conducted in SL to protect its ecosystem health and function, mainly focusing on the content and spatial distribution of pollutants in sediments, including heavy metals and forms of phosphorus (Gao et al., 2009, 2010, 2013; Shao et al., 2015; Zhang et al., 2017). Although organic and inorganic contaminants in SL have been comprehensively investigated, the microorganisms and possible biochemical reactions involved are poorly understood. Furthermore, recent studies have provided evidence that populations of diazotrophic sulfate-reducing bacteria and archaea such as specific subclades of *Woesearchaeota* and *Bathyarchaeota*, as well as methanogens, more frequently occur in surface sediments colonized by *Z. marina* seagrass meadows than in bare sediments (Sun et al., 2015; Zheng et al., 2019). This may be related to the fact that *Z. marina* seagrass meadows can provide more bioavailable organic matter.

The effects of organic phosphorus on eutrophication have been well-studied with respect to bacteria such as cyanobacteria (McMahon and Read, 2013; Dong et al., 2016; Xie et al., 2019). However, less attention has been given to the effect of OP on methanogenic microorganisms in seagrass systems. Whether OP mineralization and its final products, IP, affect methane production has not been investigated in marine environments (Zhong et al., 2017). Here, we selected different sediment layer samples of the *Z. marina* seagrass-vegetated and unvegetated regions in SL to investigate the effects of OP on methylotrophic methanogenesis, and the interaction between OP mineralization and methane production. Moreover, pure cultures of methylotrophic methanogens were further tested for OP. These results will help improve our understanding of the recycling mechanisms of organic carbon and phosphorus in methanogenic archaea in coastal wetlands and offshore environments.

## Materials and Methods

### Sediment Samples

**FIGURE 1:**
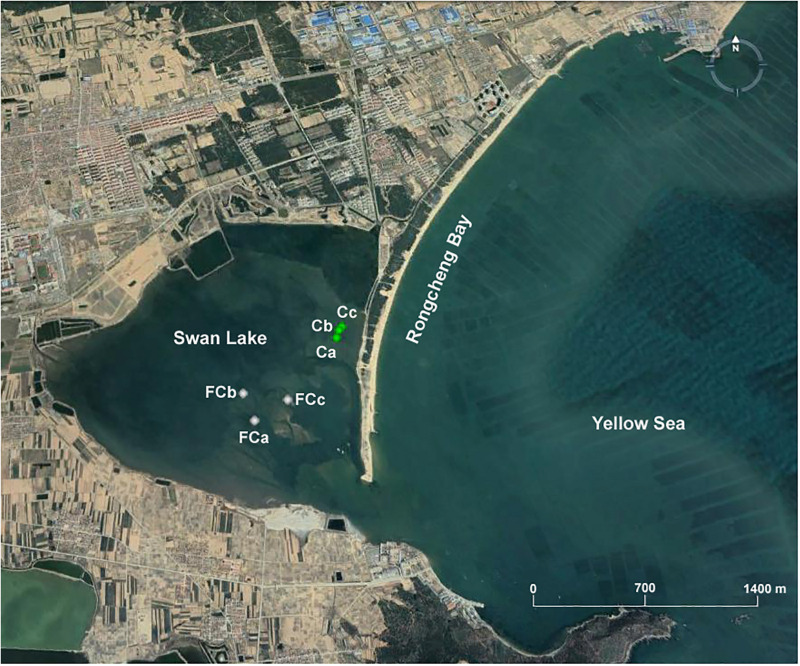
Location of sampling points in Swan Lake including three seagrass vegetated sites (Ca, Cb, Cc) and three unvegetated sites (FCa, FCb, FCc).

Marine sediment samples were collected in November 2018 from Swan Lake (37°19′N–37°22′N, 122°33′E–122°34′E), which is located in the southwestern part of Rongcheng Bay, Shandong Peninsula, northern China and connects to the Yellow Sea by means of a narrow inlet. Different sediment layers, referred to as the surface layer (0–20 cm), the middle layer (20–40 cm), and the bottom layer (40–60 cm), were collected from three seagrass-vegetated sites (Ca, Cb, Cc; 37°21′1″N-37°21′4″N, 122°34′41″E–122°34′43″E) and three unvegetated sites (FCa, FCb, FCc; 37°20′40″N–37°20′47″N, 122°34′12″E-122°34′25″E) (Figure 1). A total of 18 sediment samples were collected with a custom-made corer (inner diameter, 10 cm) during low tide. After collection, the samples were stored at 4°C for enrichment cultures and immediately transported to the laboratory under anoxic conditions.

### Enrichment of Methylotrophic Archaea

Enrichment was initiated immediately after transportation. The marine enrichment medium was natural seawater from Swan Lake, first passed through 0.45-μm filters, and then 0.22-μm filters. The filtered water was amended with 33 mmol L^–1^ trimethylamine for enrichment incubation. Before autoclaving, the medium was completely purged of O_2_ in solution and the headspace was purged with N_2_/CO_2_ (80/20, v/v). Subsequently, a 10% (w/v) sample of the sludge sample was inoculated into anaerobic tubes containing 10 ml liquid medium. All culture setup was performed in a Coy anaerobic chamber and incubated at 30°C in the dark without shaking for 1 month.

### Phytate Phosphorus Supplementation Experiments

Sodium phytate (Sigma-Aldrich, United States) at two different concentrations (2 mg L^–1^: 2P and 5 mg L^–1^: 5P) was used as the source of OP for the main experiment. Sodium phytate was additionally added to the anaerobic marine enrichment medium before inoculation for the enrichment of methylotrophic archaea.

The effect of 5 mg L^–1^ sodium phytate on pure methylotrophic methanogens was tested. *Methanosarcina mazei* was isolated from marine sediments from SL and stored in our laboratory. The methods for isolation and identification of *M. mazei* have been previously described (Wang et al., 2019). Wild-type *Methanolobus psychrophilus* strain R15 was obtained from Prof. Xiuzhu Dong’s laboratory culture collection at the Institute of Microbiology, Chinese Academy of Sciences (Zhang et al., 2008). These strains were cultivated in marine MS methanogenic media with trimethylamine as the electron donor. After three generations, the cultures were inoculated into marine MS methanogenic media containing 5 mg L^–1^ sodium phytate. Each set of experiments was conducted in triplicate.

### Analytical Methods

Methane accumulation in the headspace of the incubations was measured using a gas chromatograph (GC) 7820A (Agilent Technologies, United States) equipped with a flame ionization detector.

Available phosphorus in all enrichment cultures was analyzed using the molybdenum stibium anti-color method and quantified at 880 nm using a UV–Vis spectrophotometer as previously described (Lu, 1999).

The protein concentrations of cells in enrichment cultures were determined using the BCA method according to manufacturer’s protocol (Solarbio, China).

### Molecular Analyses

As methane production reached its plateau in each anaerobic enrichment culture, total nucleic acids were extracted using a bead-beating protocol (Shrestha et al., 2009). The extract was treated with gDNA Eraser (TaKaRa) to remove coextracted DNA, then was confirmed to be DNA-free the absence of PCR products using the universal primers Ba27f/Ba907r for bacteria, and Ar109f/Ar915r for archaea. Reverse transcription was performed after RNA denaturation at 70°C for 10 min, followed by an incubation step at 42°C for 50 min according to the instructions in the PrimeScript II 1st strand cDNA Synthesis Kit (TaKaRa). To assess archaeal community composition, 16S rRNA of the V4–V5 distinct region was amplified using the specific primers Ar519f and Ar915r from community cDNA from the enrichment cultures and sequenced using high-throughput sequencing on an Illumina Hiseq2500 platform.

Sequence analyses were performed using USEARCH software (version 10)^1^ after sequencing data processing. The most frequently occurring sequence was extracted as a representative sequence for each OTU, defined using a threshold level of 97% identity. For each representative sequence, the SILVA database was used to annotate taxonomic information. The average relative abundance (%) of the predominant genus-level taxon in each sample was calculated by comparing the number of assigned sequences at the genus classification level with the total number of obtained sequences.

### Accession Number of Nucleotide Sequences

Raw sequence reads of the archaeal 16S rRNA gene sequences have been deposited in the Sequence Read Archive at the National Center for Biotechnology Information under accession nos. SRR11591982-SRR11592011.

## Results

### Enrichment Cultivation of Methylotrophic Archaea From Different Sites

To selectively enrich for methylotrophic archaea, especially methanogenic archaea in the different ecosystems of SL, trimethylamine-amended enrichment cultures were established for sediment samples from different sediment layers from the seagrass-vegetated and unvegetated regions of the lake. In all anaerobic incubation experiments, methane was produced after a short lag time from six sites at three depths (Supplementary Figure S1). Similar methanogenic indications were observed in vegetated/unvegetated regions, except for FCc sites where methane production from samples in the surface (s) and middle (m) layers was much higher than in the bottom (b) layer (Supplementary Figure S1).

### Impact of Phytate Phosphorus on Methanogenesis Performance at Different Sediment Depths

**FIGURE 2:**
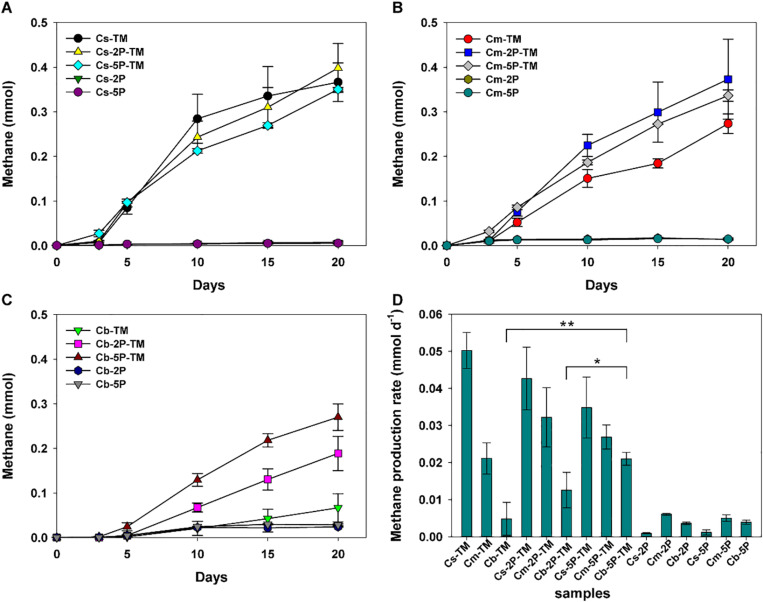
Methane production **(A–C)** and methane production rate **(D)** from enrichments of three sediment depths (s, m, b) in vegetated regions with and without addition of phytate phosphorus (2P and 5P). TM, trimethylamine. Data on methane production rates were tested for significant differences using a *t*-test (*p < 0.05, **p < 0.01). Data are the means and SDs for triplicate cultures.

**FIGURE 3:**
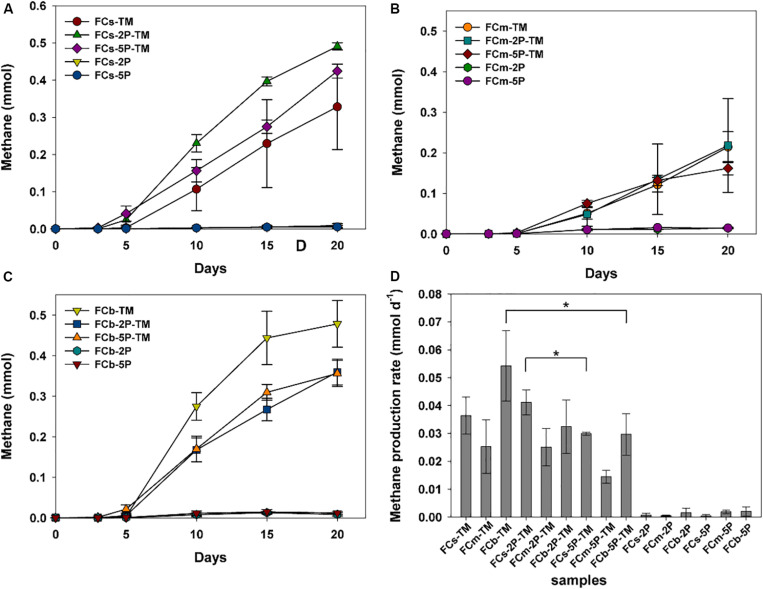
Methane production **(A–C)** and methane production rate **(D)** from enrichments of three sediment depths (s, m, b) in unvegetated regions with and without addition of phytate phosphorus (2P and 5P). Data on methane production rates were tested for significant differences using a *t*-test (*p < 0.05). Data are the means and SDs for triplicate cultures.

Methylotrophic archaea enriched from the s, m, and b sediment layers in vegetated/unvegetated regions respectively were mixed in equal proportions as inocula (vegetated regions: Cs, Cm, and Cb; unvegetated regions: FCs, FCm, and FCb) to compare the effect of phytate phosphorus (2P and 5P) on methanogenesis. Methane increased to 0.068–0.491 mmol over 20 days of incubation in all trimethylamine added experiments. Methane generation was limited in experiments where only phytate phosphorus was added (Figure 2). Compared with samples without addition of phytate phosphorus, both 2P and 5P promoted methane production in the Cm and Cb layers of vegetated regions by 37% (from 0.27 to 0.37 mmol) for 2P and 303% (from 0.067 to 0.270 mmol) for 5P (Figures 2A–C). In contrast, in unvegetated regions, phytate phosphorus enhanced methane production by 48% (from 0.33 to 0.49 mmol) in the FCs layer, but inhibited production in the FCb layer by 25% (from 0.48 to 0.36 mmol) (Figures 3A–C).

Different methane production rates were found at different depths in both regions (Figures 2D, 3D). To be specific, in vegetated regions, without adding phytate phosphorus, the Cs layer had the highest methane production rate (0.05 ± 0.005 mmol d^–1^), as much as 2.4- and 10.4-fold higher than that of Cm and Cb layers, respectively. When 2P and 5P were added, a similar tendency to high methane production rates was observed. The highest methane production rate in the Cs layer was 1.6- and 3.4-fold higher than in the Cm and Cb layers (*p* < 0.05), respectively. However, the addition of phytate phosphorus had no significant effect on the methane production rate in the Cs and Cm layers (*p* > 0.05), but significantly accelerated the methane production rate by 4.3-fold in the Cb layer (*p* = 0.004, *p* < 0.01), compared with the experiments without adding phytate phosphorus (Figure 2D). In contrast, in unvegetated regions, the FCb had the highest methane production rate of 0.054 ± 0.013 mmol day^–1^, which was 2- and 1.5-fold higher than that of the FCm and FCs layers, respectively. No significant difference (*p* > 0.05) in the methane production rate was found in the FCs and FCm layers between treatments with and without the addition of phytate phosphorus. However, phytate phosphorus significantly inhibited the methane production rate by 1.8-fold in the FCb layer (*p* = 0.04, *p* < 0.05) (Figure 3D).

### Available Phosphorus Generation

**FIGURE 4:**
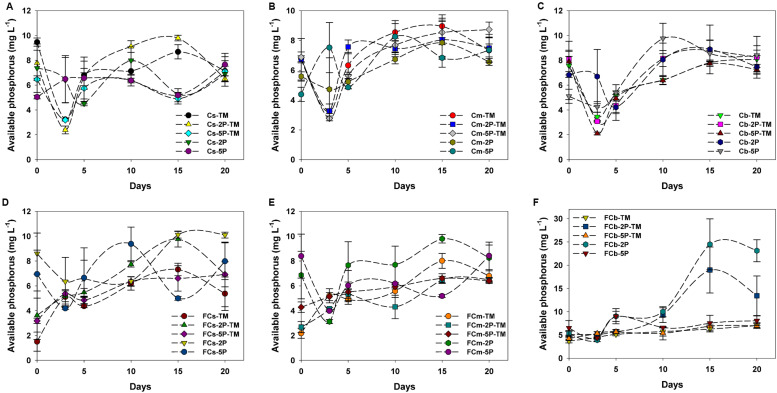
Concentration of available phosphorus from enrichments of different sediment depths in vegetated **(A–C)** and unvegetated **(D–F)** regions with and without addition of phytate phosphorus (2P and 5P). Data are the means and SDs for triplicate cultures.

Overall, concentrations of available phosphorus showed obvious fluctuations in treatments with and without the addition of phytate phosphorus (Figure 4). No available phosphorus was detected in anaerobic marine enrichment media without sediment inocula (data not shown). In vegetated areas, in trimethylamine-amended enrichment samples with and without the addition of phytate phosphorus, the concentrations of available phosphorus showed significant fluctuations from day 1 to day 5, decreased over the subsequent 3 days, then increased (Figures 4A–C). In contrast, the concentration of available phosphorus was stable or increased in the corresponding treatment samples from unvegetated regions during the enrichment period (Figures 4D–F). Unlike samples from vegetated regions with only the addition of phytate phosphorus, in unvegetated regions the concentration of available phosphorus decreased within the first 3 days of enrichment (Figures 4D–F). However, the concentration of available phosphorus significantly increased after 10 days in the 2P added samples such as FCb-2P and FCb-2P-TM from the unvegetated regions (Figure 4F).

### Variability in Biomass Levels

**FIGURE 5:**
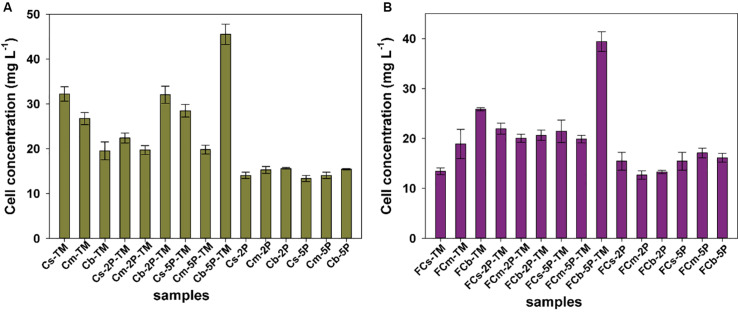
The protein concentration of total cells from enrichments of different sediment depths in vegetated **(A)** and unvegetated **(B)** regions with and without addition of phytate phosphorus (2P and 5P). Data are the means and SDs for triplicate cultures.

Biomass of microorganisms was assayed at the end of incubation. The total amount of biomass in sediments from vegetated regions was higher than that in unvegetated regions, a 1.2-fold increase in samples with trimethylamine and 1.1-fold in samples with addition of phytate phosphorus, respectively (Figure 5). The results further showed that phytate phosphorus did not increase the biomass in the Cs layer in vegetated regions, but significantly enhanced the growth of microorganisms in the Cb layer (Figure 5A). In unvegetated regions, phytate phosphorus slightly increased biomass accumulation in the FCs layer, but significantly increased biomass in the FCb layer with the addition of 5P (Figure 5B).

### Methylotrophic Methanogens in Enrichment Cultures

**FIGURE 6:**
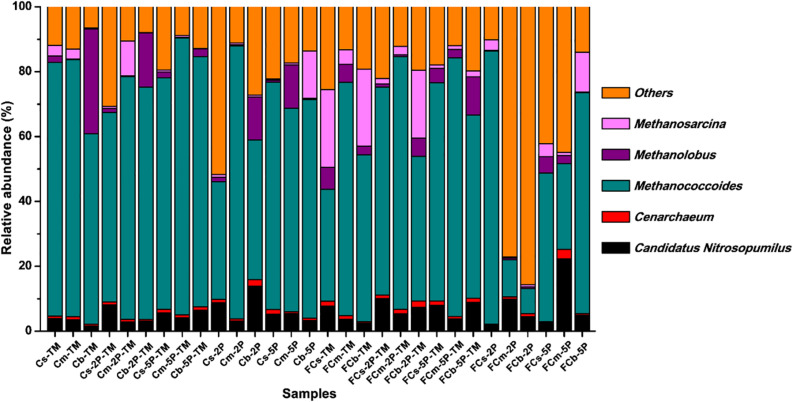
Relative abundance of archaea from enrichments of different sediment depths in vegetated and unvegetated regions with and without addition of phytate phosphorus (2P and 5P) based on 16S rRNA gene amplicon sequencing.

The diversity of archaea, especially methylotrophic methanogens, in our enrichment cultures was investigated using high-throughput sequencing from community cDNA based on an archaeal 16S rRNA gene (Figure 6). Among the archaeal community, methylotrophic methanogens including *Methanococcoides*, *Methanosarcina*, and *Methanolobus* dominated in enrichment cultures. *Methanococcoides* was most abundant in the enriched samples except for FCm-2P and FCb-2P, and its relative abundance ranged from 26.4 to 85.4%. *Methanosarcina* was mainly found in unvegetated regions, with a maximum abundance of 24.0% in FCs-TM and FCb-TM samples. The total relative abundance of *Methanosarcina* in unvegetated regions was higher than in vegetated regions. In contrast to *Methanosarcina*, the total relative abundance of *Methanolobus* in vegetated regions was higher than that of unvegetated regions, with a maximum relative abundance of 32.4% in the Cb-TM sample. However, the relative abundance of *Methanolobus* in the Cb layer from vegetated regions was significantly reduced by the addition of phytate phosphorus, and higher than that of the sample without phytate phosphorus. In unvegetated regions, the addition of phytate phosphorus significantly reduced the relative abundance of *Methanosarcina* in the FCb layer.

### Effect of Phytate Phosphorus on *Methanolobus* and *Methanosarcina*

**FIGURE 7:**
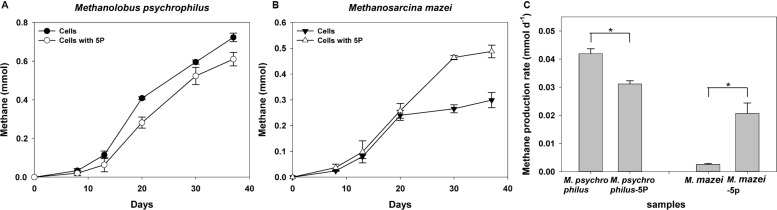
Methane production of *Methanolobus psychrophilus* and *Methanosarcina mazei* with the addition of 5 mg L^–1^ sodium phytate (5P). Time courses for methane production of *M. psychrophilus* **(A)**. Time courses for methane production of *M. mazei* **(B)**. Methane production rate of *M. psychrophilus* and *M. mazei* **(C)**. Data on methane production rates were tested for significant differences using a *t*-test (*p < 0.05). The error bars represent SDs from the mean for triplicate cultures.

We further investigated the effect of phytate phosphorus on pure methanogenic strains such as *Methanolobus* and *Methanosarcina*, the characteristic methanogenic species in both vegetated and unvegetated regions. Methane was produced by all cultures of both methanogens with trimethylamine as the substrate (Figure 7). During a 37-day incubation, phytate phosphorus had a distinct effect on both *Methanolobus psychrophilus* and *Methanosarcina mazei*. Approximately 12.0–44.7% inhibition of the methane production *M*. *psychrophilus* was observed for the first 8 days compared with cells without the addition of phytate phosphorus (Figure 7A). An inhibitory effect of phytate phosphorus was also observed affecting the maximum methane production rate of *M*. *psychrophilus*, which was inhibited by 25.6% in 20 days (*p* = 0.02, *p* < 0.05) (Figure 7C). In contrast, the same concentration of phytate phosphorus significantly promoted methane production by *M*. *mazei* by 7.5–75.2% compared with cells without added phytate phosphorus (Figure 7B). Phytate phosphorus increased the maximum methane production rate of *M*. *mazei* by up to 716% at 30 days (*p* = 0.02, *p* < 0.05) (Figure 7C).

## Discussion

To our knowledge, our study was the first to investigate the effects of OP on methylotrophic methanogenesis in coastal lagoon sediments. Our results show that there was a significantly differentiated effect on methanogenesis performance from the addition of phytate phosphorus detected at different depths of the two sediment types. Phytate phosphorus significantly promoted methane production in the bottom layer of sediments in the vegetated regions, whereas in the unvegetated regions, phytate phosphorus inhibited methane production in the bottom layer of sediments. However, the results also demonstrate that phytate phosphorus had less effect on methane production in the surface and middle layers of sediments in both regions.

These findings are consistent with our knowledge that the surface layer sediments colonized by the seagrass *Z. marina* provide more bioavailable organic matter (Sun et al., 2015; Zheng et al., 2019), such as methylated compound, that serve as a substrate for methanogens. Methylotrophic methanogens were able to outcompete SRB by virtue of excess substrate by which they maintained higher activity in the surface layer sediments; thus, it was reasonable that phytate phosphorus would not affect methane production to a great extent. In contrast to the surface layer sediments, the bottom layer sediments lack some essential substances for microbial life in Arai et al. (2016), so methylotrophic methanogens may not be unfavored for methane production with increasing sediment depths (Lee et al., 2015). However, when phytate phosphorus was added to the bottom layer sediments, methane production rate was significantly increased, meaning that OP may be a source of phosphorus rather than the substrate for methanogens in deeper sediments (Broman et al., 2017). In contrast to vegetated regions, the unvegetated regions, lacking seagrass meadow colonization, were clearly divided into sulfate zones, sulfate-methane transition zones, and methane zones. These regions featured an intermediate transition zone with less organic matter, such as the middle layer, and the total numbers and diversity of methanogens were further reduced, possibly owing to competition with SRB for substrates (Kevorkian et al., 2018; Glombitza et al., 2019). As a result, the addition of phytate phosphorus did not significantly change the competitive relationship between methylotrophic methanogens and SRB. The methanogenic bottom layer was more favorable for methanogenesis, but the addition of phytate phosphorus inhibited the methane production of methylotrophic methanogens, suggesting that the addition of phytate phosphorus may affect the boundary between layers in the sediments in unvegetated regions.

**FIGURE 8:**
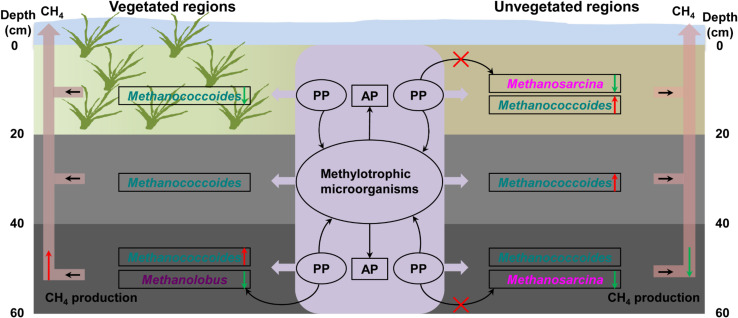
Illustration of the mechanism underlying the effect of organic phosphorus on methylotrophic methanogenesis. PP, phytate phosphorus; AP, available phosphorus. The red and green arrows indicate methane production.

OP mineralization provides a potential source of bioavailable phosphorus for microbial growth in coastal wetland sediments (Bai et al., 2019). In treatments with trimethylamine but without addition of phytate phosphorus, the concentration of available phosphorus exhibited significant fluctuations, indicating that inoculated methylotrophic microorganisms such as phosphorus-solubilizing bacteria greatly promoted the release of OP from sediments (Broman et al., 2017). The addition of phytate phosphorus significantly promoted the growth of methylotrophic methanogens, increasing methane production in the bottom layer in the vegetated regions. However, phytate phosphorus promoted the growth of other methylotrophic microorganisms in the bottom layer in the unvegetated regions, rather than methylotrophic methanogens, an observation consistent with less methane production in these regions. This further suggested that methylotrophic microorganisms from the enrichment of sediments promoted the production of available phosphorus in the vegetated and unvegetated regions. Increased available phosphorus may support methylotrophic methanogens’ facilitation of phosphorus metabolism (Figure 8).

As expected, methylotrophic methanogens predominated in the archaeal community enriched with trimethylamine. *Methanococcoides* (Franzmann et al., 1992), *Methanolobus* (Konig and Stetter, 1982), and *Methanosarcina* (Sowers et al., 1984) were highly active. Therefore, it seems that trimethylamine may be an important substrate for these methylotrophic methanogens in sediments. The difference in relative abundance between *Methanolobus* and *Methanosarcina* in the vegetated and unvegetated regions may be correlated with the vegetation of seagrass meadows (Zhang et al., 2008), where a large quantity of organic compounds such as choline and glycine betaine can be exuded from the roots of seagrasses and converted into methylated compounds instead of hydrogen that can be easily used by *Methanolobus* (Zhang et al., 2008; Lyimo et al., 2009), rather than for the growth of *Methanosarcina*. However, the higher relative abundance of *Methanosarcina* in sediments in the unvegetated regions suggested that H_2_ may be an important source of reducing power for the growth of *Methanosarcina*, in agreement with previous reports that H_2_ and CO_2_ directly contributed to methanogenesis in marine sediments (Parkes et al., 2007; Jorgensen and Parkes, 2010; Beulig et al., 2018; Glombitza et al., 2019). The contribution of methylated compounds to methane production in offshore marine environments with different vegetation types may need to be assessed.

To complement the complex environment studies, experiments with pure cultures tested the hypothesis that phytate phosphorus inhibited methane production of *M. psychrophilus* but enhanced that of *M. mazei*. These results are consistent with the inhibitory effect of phytate phosphorus on *Methanolobus* species in the deeper seagrass (*Z. marina*)-vegetated sediments, but were contrary to the same effect on *Methanosarcina* species in the unvegetated sediments. This can be explained by phytate phosphorus being inhibitory to the growth of specific methylotrophic *Methanolobus* species. In contrast, the multitrophic *Methanosarcina* species may use phytate phosphorus as a stimulatory factor to promote methyl metabolism to produce methane, but its growth was vulnerable to be inhibited by other microorganisms in the natural environment. Therefore, it seems that OP or available phosphorus from OP mineralization had a selective effect on different methanogens in vegetated and unvegetated systems (Figure 8). This new finding may help to explain the different effects of OP on methanogens in the deeper bare sediments, with the activities of other methylotrophic microorganisms enhanced by OP to compete for substrates with methylotrophic methanogens. In addition, *Candidatus_Nitrosopumilus*, *Cenarchaeum*, and other unknown archaea were also dominant in samples with the addition of OP, suggesting these archaea might participate in the OP cycle. However, further investigation of the real ecological function is needed.

## Data Availability Statement

The datasets generated for this study can be found in NCBI, with accession number PRJNA627294.

## Author Contributions

SZ designed and performed the experiments and drafted the manuscript. BW and GX performed the experiments. FL designed the study and revised the manuscript. All authors contributed to the article and approved the submitted version.

## Conflict of Interest

The authors declare that the research was conducted in the absence of any commercial or financial relationships that could be construed as a potential conflict of interest.

## Footnotes

1 http://www.drive5.com/usearch/

## Supplementary Material

FIGURE S1 Methane production from enrichment of three sediment depths (surface, s; middle, m; and bottom layer, b) from different sites (a, b, c) of vegetated (C) **(A–C)** and unvegetated (FC) **(D–F)** regions. Data are the means and SDs for triplicate cultures.
