# Supplementary figures and images for: Effects of Organic Phosphorus on Methylotrophic Methanogenesis in Coastal Lagoon Sediments With Seagrass (Zostera marina) Colonization
Source: Front Microbiol. 2020 Jul 31;11:1770. doi: 10.3389/fmicb.2020.01770 (PMC7411354; doi:10.3389/fmicb.2020.01770)

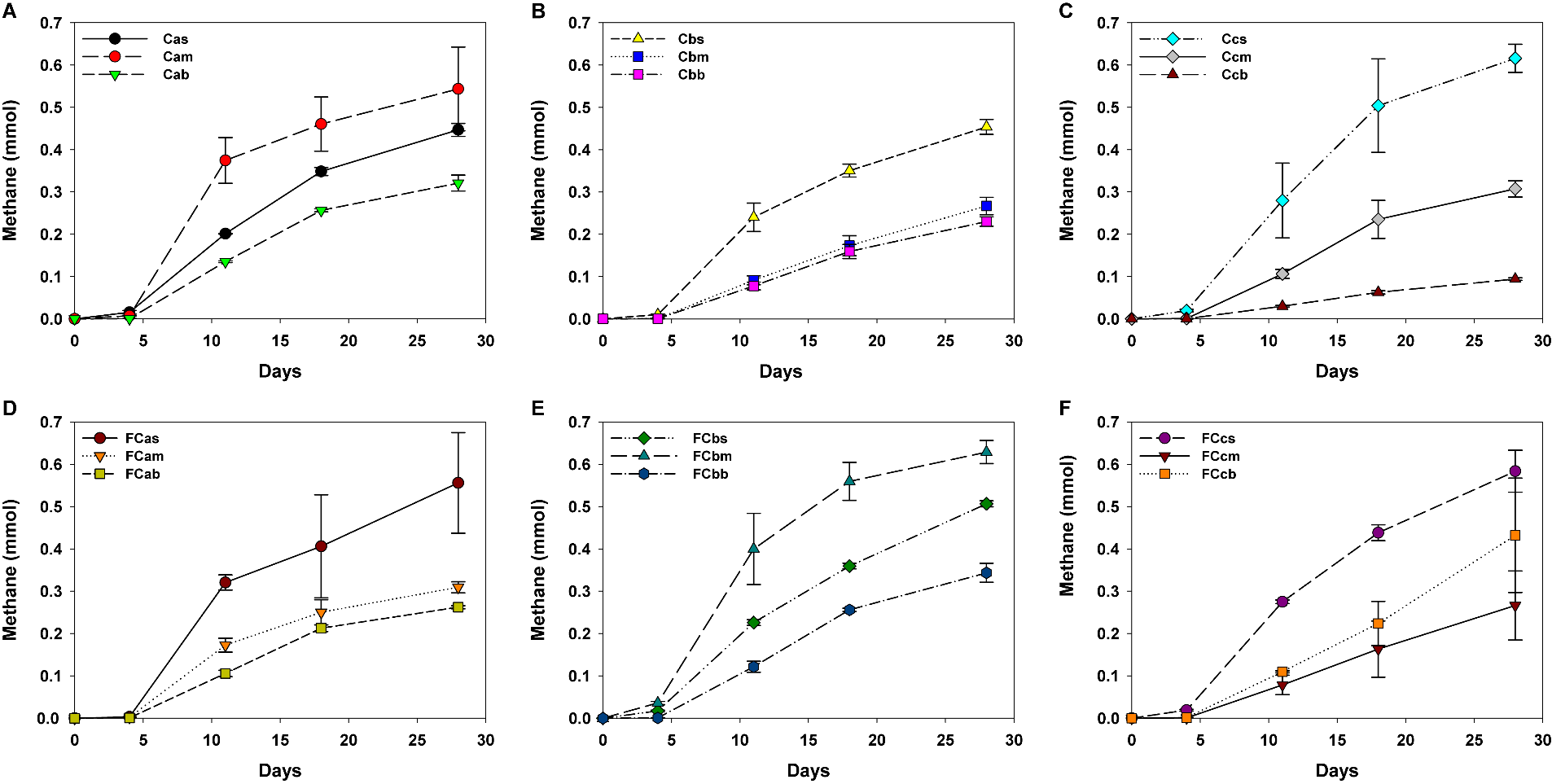

Supplement: FIGURE S1 — Methane production from enrichment of three sediment depths (surface, s; middle, m; and bottom layer, b) from different sites (a, b, c) of vegetated (C) (A–C) and unvegetated (FC) (D–F) regions. Data are the means and SDs for triplicate cultures. [file Image_1.tif]
